# Multi-model Hydroclimate Projections for the Alabama-Coosa-Tallapoosa River Basin in the Southeastern United States

**DOI:** 10.1038/s41598-020-59806-6

**Published:** 2020-02-18

**Authors:** Sudershan Gangrade, Shih-Chieh Kao, Ryan A. McManamay

**Affiliations:** 1 The Bredesen Center, University of Tennessee, Knoxville, TN 37996 USA; 2 Climate Change Science Institute, Oak Ridge National Laboratory, Oak Ridge, TN 37831 USA; 3 Environmental Sciences Division, Oak Ridge National Laboratory, Oak Ridge, TN 37831 USA; 4 Department of Environmental Science, Baylor University, Waco, TX 76706 USA

**Keywords:** Climate sciences, Hydrology

## Abstract

This study uses a high-resolution, process-based modeling framework to assess the impacts of changing climate on water resources for the Alabama-Coosa-Tallapoosa River Basin in the southeastern United States. A 33-member ensemble of hydrologic projections was generated using 3 distributed hydrologic models (Precipitation-Runoff Modeling System, Variable Infiltration Capacity, and Distributed Hydrology Soil Vegetation Model) of different complexity. These hydrologic models were driven by dynamically downscaled and bias-corrected future climate simulations from 11 Coupled Model Intercomparison Project Phase 5 global climate models under Representative Concentration Pathway 8.5 emission scenario, with 40 years each in baseline (1966–2005) and future (2011–2050) periods. The hydroclimate response, in general, projects an increase in mean seasonal precipitation, runoff, and streamflow. The high and low flows are projected to increase and decrease, respectively, in general, suggesting increased likelihood of extreme rainfall events and intensification of the hydrologic cycle. The uncertainty associated with the ensemble hydroclimate response, analyzed through an analysis of variance technique, suggests that the choice of climate model is more critical than the choice of hydrologic model for the studied region. This study provides in-depth insights of hydroclimate response and associated uncertainties to support informed decisions by water resource managers.

## Introduction

A changing climate is projected to intensify regional and global hydrologic cycles^1,2^. These alterations in hydrologic cycles will potentially increase the frequency and magnitudes of hydroclimate extremes such as floods and droughts, and potentially impact water resource availability due to changes in the seasonality of streamflow and runoff^3–5^. The future hydrologic projections are, therefore, important to inform mitigation and adaptation strategies aimed at addressing impacts of climate change in addition to increasing water demands. Moreover, reliable estimates of hydroclimate extreme trends can ensure better preparedness of society and infrastructure from threats arising from extreme events and their socioeconomic impacts^6^.

Studies assessing climate change impacts on future hydrology at regional or catchment scales often adapt a standard procedure involving the use of a hierarchical hydro-meteorological framework (hereinafter “modeling framework”), including selection of the following key elements: (a) greenhouse gas emission scenario, (b) global climate model (GCM), (c) downscaling method (statistical or dynamical), (d) bias correction of downscaled data (if required), and (e) hydrologic model^7–10^. These hydrologic projections are inevitably associated with uncertainties introduced at each stage of the modeling framework. In addition to external factors such as natural variability and the choice of emission scenarios, many uncertainties are model-related, such as model assumptions, structures, accuracy, initial conditions, calibration procedures, training datasets, and the spatial and temporal scales of implementation^7,11,12^. An ideal but non-pragmatic way to characterize these uncertainties would encompass producing ensemble hydroclimate projections using a complete sample of uncertainty sources. However, given the limited resources, most impact assessment studies can focus only on a subset of these choices, thereby resulting in underestimation of the uncertainties in hydroclimate projections^7,13^.

Given the plethora of choices in the above-mentioned modeling framework and their significance in hydroclimatic projections, many studies have investigated the effects of individual sources of uncertainties^14–16^, as well as combined uncertainties due to different methodological choices within the modeling framework^7,8,17–23^. Several studies at global and regional scales indicate that the uncertainties from climate models are a more important source of uncertainty than other factors such as greenhouse gas emission scenarios and hydrologic model structures^8,12,19,24^. On the other hand, Bosshard *et al*. (2013) revealed that the prominent sources of uncertainty vary by season in the Alpine region, where uncertainties arising from climate models dominate during summer and fall, whereas choices of statistical processing methods and hydrologic models are more prevalent during winter and spring^7^. Similarly, Chegwidden *et al*. (2019) demonstrated that choices of GCM and greenhouse emission pathways are the dominant contributors to annual streamflow volume, and the choices of hydrologic model and parameters are prominent in capturing low-flow uncertainties over the US Pacific Northwest^23^. While multiple climate models and greenhouse gas emission scenarios have been used to capture the ensemble of climate scenarios in the past two decades, such studies are often limited to the choice of a single hydrologic model^17,20,22,25–27^. Despite studies indicating that choice of hydrologic model can produce substantial differences in hydrologic projections, at times exceeding the mean signal from climate scenarios^11^, the use of multiple hydrological models has only begun to gain traction^21,28,29^.

The selection of appropriate hydrologic model(s) in the modeling framework remains a challenge, as decisions so subjective in nature require careful consideration of several factors, including model applicability, suitable spatiotemporal scale of implementation, availability of computational resources, quality of meteorological forcings and land surface parameters, and the overall technical feasibility^30^. While certain applications—such as hydrodynamic modeling applied at watershed scales—warrant fine-scale outputs from hydrologic modeling (<100 m)^31^, the scalability of these implementations at regional scales is an obvious challenge. Studies have demonstrated that lumped or coarse-scale semi-distributed hydrologic models may yield similar hydroclimate projections compared to fine-scale hydrologic models^30^. However, a more elaborate comparison among models with very distinct spatial scales and structures is still lacking.

The goal of this study is to assess the impacts of changing climate on water resources through multi-model ensemble hydroclimate projections for the Alabama-Coosa-Tallapoosa (ACT) River Basin in the southeast United States (SEUS). While SEUS is considered “water-rich,” water allocation conflicts within two major river basins including ACT have created a political issue between the states of Georgia, Alabama, and Florida. The increasing water demand due to population growth and urbanization is further likely to deepen water stress in the future^32^. In addition, the SEUS is relatively underrepresented in the existing climate impact assessments on hydrology^33^. While some studies at the regional scale have been conducted, this study aims to provide a more comprehensive, ensemble-based hydroclimate evaluation over the ACT River Basin.

Overall, the main objectives of this study are to (a) develop an ensemble of high-resolution hydroclimate projections for the ACT River Basin using multiple climate and hydrologic models, and (b) analyze the relative uncertainty contribution between climate and hydrologic models. To accomplish these objectives, a hierarchical multi-model framework with process-based hydro-meteorological models over the ACT River Basin was used. An ensemble of 33 hydroclimate projections using a combination of 11 GCMs and 3 distinct hydrologic models was produced for 1966–2005 baseline and 2011–2050 future periods under the Representative Concentration Pathway 8.5 (RCP8.5) scenario. Various hydrologic indices including long-term seasonal mean, high, and low streamflow were investigated and the effects of various sources of uncertainties were analyzed. Through the incorporation of a high-resolution modeling framework, this assessment is expected to provide fine-scale ensemble hydroclimate projections to support local stakeholders, including water resource managers from 15 large reservoirs and city planners from several urban areas (including Atlanta), for more informed decisions.

## Study Area

**Figure 1:**
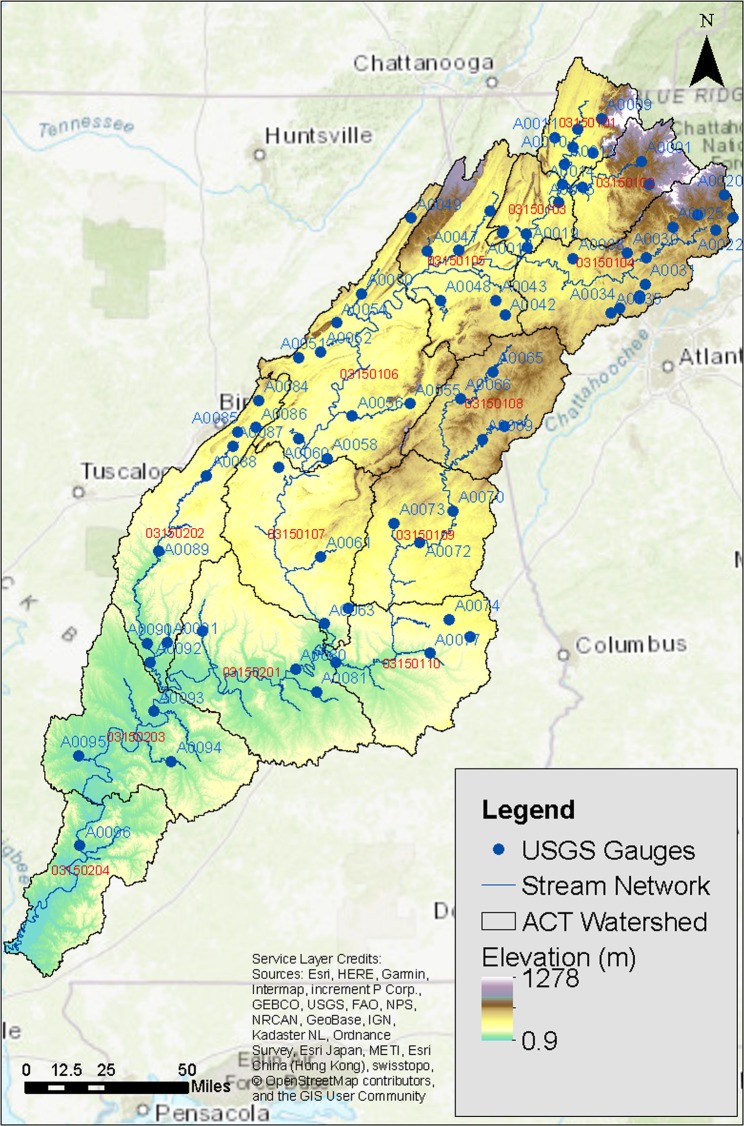
The study area showing the Alabama-Coosa-Tallapoosa River Basin along with major stream networks and USGS gauges used in analysis. The figure was created using ESRI ArcMap (https://desktop.arcgis.com/en/arcmap/) Version 10.6.1.9270. The base map used in the figure is obtained from OpenStreetMap and attributed in the service layer credits on the figure “© OpenStreetMap contributors”.

The study area consists of the ACT River Basin covering the northeastern and east-central parts of Alabama, northwestern Georgia, and small parts of Tennessee (Fig. 1). The ACT River Basin, classified as a US Hydrologic Subregion (HUC04 = 0315), has an approximate drainage area of 59,100 km^2^ and includes 14 US Hydrologic Subbasins (HUC08s). The Alabama River is formed by the confluence of the Coosa and Tallapoosa Rivers near Montgomery, AL. The Coosa River flows through HUC08s 03150101 to 03150107 while the Tallapoosa River flows through HUC08s 03150108 to 03150110. The subregion has a relatively flat topography with a small mountainous region in the north. Elevation ranges from sea level to 1278 m based on the National Elevation Dataset^34^. The soil type consists mainly of sandy loam and silty loam. The ACT River Basin receives an annual average of 1379 mm of precipitation primarily from rainfall with minimal influence of snow on runoff. Forest is the major landcover type in the ACT River Basin, which results in high evapotranspiration ranging from 762–1067 mm (56–78% of annual precipitation), generally increasing from north to south. The study area includes 15 large reservoirs including 5 federal dams^35^. The major urban areas in the ACT River Basin include suburban areas of Atlanta (Kennesaw, GA and Marietta, GA); Birmingham, AL; Montgomery AL; and Mobile, AL. The selected US Geological Survey (USGS) streamflow gauges used in the study (assigned a five-character unique ID for brevity; a reference table is provided in supplementary material (SI) Table S2) and HUC08s are marked on Fig. 1 for reference.

## Data and Methods

### General modeling framework

Various choices available along the hierarchical hydro-meteorological modeling chain necessitate an evaluation of all potential options when designing a climate change impact assessment study. The modeling framework employed in this study mainly constituted two elements contributing to uncertainty: (1) downscaling of coarse resolution GCM data to the regional scale and (2) using regional scale meteorological forcings to drive calibrated hydrologic models. This modeling framework is employed after careful consideration of the goals of the study, availability of computational resources, time constraints, and stakeholder needs. A multi-model ensemble of hydrologic projections was created using a combination of 11 climate models and 3 distinct hydrologic models. Each combination of 11 climate models and 3 hydrologic models was employed, thereby producing 33 sets of hydroclimate projections.

### Climate models

The climate projections used in this study were generated by dynamically downscaling 11 Coupled Model Intercomparison Project Phase 5 GCMs using Regional Climate Model version 4 (RegCM4) to a horizontal spatial resolution of 18 km^36^. These climate projections were further statistically bias-corrected by the Parameter-elevation Regressions on Independent Slopes Model (PRISM) meteorological data using the quantile mapping technique^37,38^. The bias correction was conducted at the monthly scale using the 1/24° (~4 km) resolution 1966–2005 monthly PRISM precipitation and temperature data as observation. The monthly correction values were then evenly distributed to daily time series of precipitation (using ratio adjustment) and temperature (using degree adjustment). The climate projections provide key meteorological forcing data such as daily precipitation, maximum and minimum daily temperature, and wind speed for hydrologic models for 40 years in the baseline period (1966–2005) and another 40 years in the future period (2011–2050). The future projections are obtained under the RCP8.5 business as usual case scenario, which assumes high population and slow income growth. Further details about regional downscaling effort is detailed in Ashfaq *et al*.^36^. A comparison of bias corrected RegCM4 outputs and their performance evaluation over ACT region is presented in the supplementary information.

### Hydrologic models

This study used three distinct hydrologic models of varying complexity and spatiotemporal resolution, including the Precipitation Runoff Modeling System (PRMS, ~7.5 km spatial resolution at daily timestep), Variable Infiltration Capacity model (VIC, ~4 km at three-hourly timestep^27^), and Distributed Hydrology Soil Vegetation Model (DHSVM, 90 m at three-hourly timestep^39^). These models were selected due to their wide range of applications in climate change studies^26,27,40–42^. In addition, these models can simulate hydrologic processes at a fine spatial resolution using distributed process-based equations allowing them to better capture meteorological and basin heterogeneity. For model calibration and validation, all three hydrologic models were implemented for the historic period of 1980–2012 using the same meteorological forcings obtained from the Daymet dataset^43^. Year 1980 was used for model spin-up. The model performance was evaluated for 62 USGS gauges across the ACT River Basin. A detailed description of these models and their calibration strategies is presented in the supplementary information.

### Hydrologic indices

The calibrated PRMS, VIC, and DHSVM models were used to generate hydroclimate projection for each of the 11 GCMs, resulting in a 33-member ensemble. The first year of meteorological data was repeated during the hydrologic simulation in both the baseline and future periods to initiate hydrologic model spin-up and has been discarded in the analysis. The outputs of hydrologic models included runoff at the aggregated HUC08 level; daily streamflow values at the gauge level for 62 selected gauges were used for analysis and comparison (Fig. 1, gauges are assigned a five-character unique ID for the sake of brevity. An association table is provided in SI Table S2).

Three types of indices were selected to evaluate changes in hydrologic response from baseline (1966–2005) to future (2011–2050) periods. They include:

- Mean seasonal percent change in precipitation (ΔP), runoff (ΔR), and streamflow (ΔQ)
- Mean percentage change in high runoff/streamflow (ΔR95/ΔQ95), where high runoff/streamflow indicates the 95th percentile runoff/streamflow statistics
- Mean percentage change in low runoff/streamflow (ΔR05/ΔQ05), where low runoff/streamflow indicates the 5th percentile runoff/streamflow statistics.

In all cases, percentage change is calculated with reference to baseline values (i.e., 100 × (future - baseline)/baseline). The following breakdown of months was used to characterize seasons: Winter (December, January, and February), Spring (March, April, and May), Summer (June, July, and August), and Fall (September, October, and November).

### Uncertainty quantification

Analysis of variance (ANOVA) was used to quantify the of relative contribution of uncertainties in hydroclimate projections arising from different sources and their interactions, similar to other studies^7,9,23,44^. Based on this technique, the total variance can be explained by the sum of variances introduced by individual components and their interactions. Since, this study focuses on two main sources of uncertainties arising from (a) 11 climate models and (b) 3 hydrologic models, the following equation was developed:

1 $${Y}_{i,j}=\mu +C{M}_{i}+H{M}_{j}+{(CM\times HM)}_{ij}+e,$$

where *Y* is the climate change indicator for the *i* climate model and the *j* hydrologic model; *μ* and *e* denote overall mean and error, respectively. The terms *CM*, *HM*, and *CM × HM* denote the relative contribution of each source of uncertainties arising from climate models, hydrologic models, and interaction of climate and hydrologic models, respectively. The analysis was performed for the hydrologic indices including ΔQ for each season, ΔQ95, and ΔQ05.

## Results and Discussion

### Model performance

The historic model performance of the hydrologic models was evaluated for two different hydrologic variables including runoff (monthly, aggregated at HUC08 level) and streamflow (both daily and monthly for 62 gauges) due to different calibration procedures for the hydrologic models. While VIC was calibrated to monthly USGS WaterWatch runoff and DHSVM was calibrated to daily streamflow at USGS gauge locations, PRMS was calibrated in a two-step fashion in which the first step involved runoff calibration at the HUC08 level to monthly USGS WaterWatch and the second step involved calibration of streamflow against USGS gauge data at the daily time scale.

**Figure 2:**
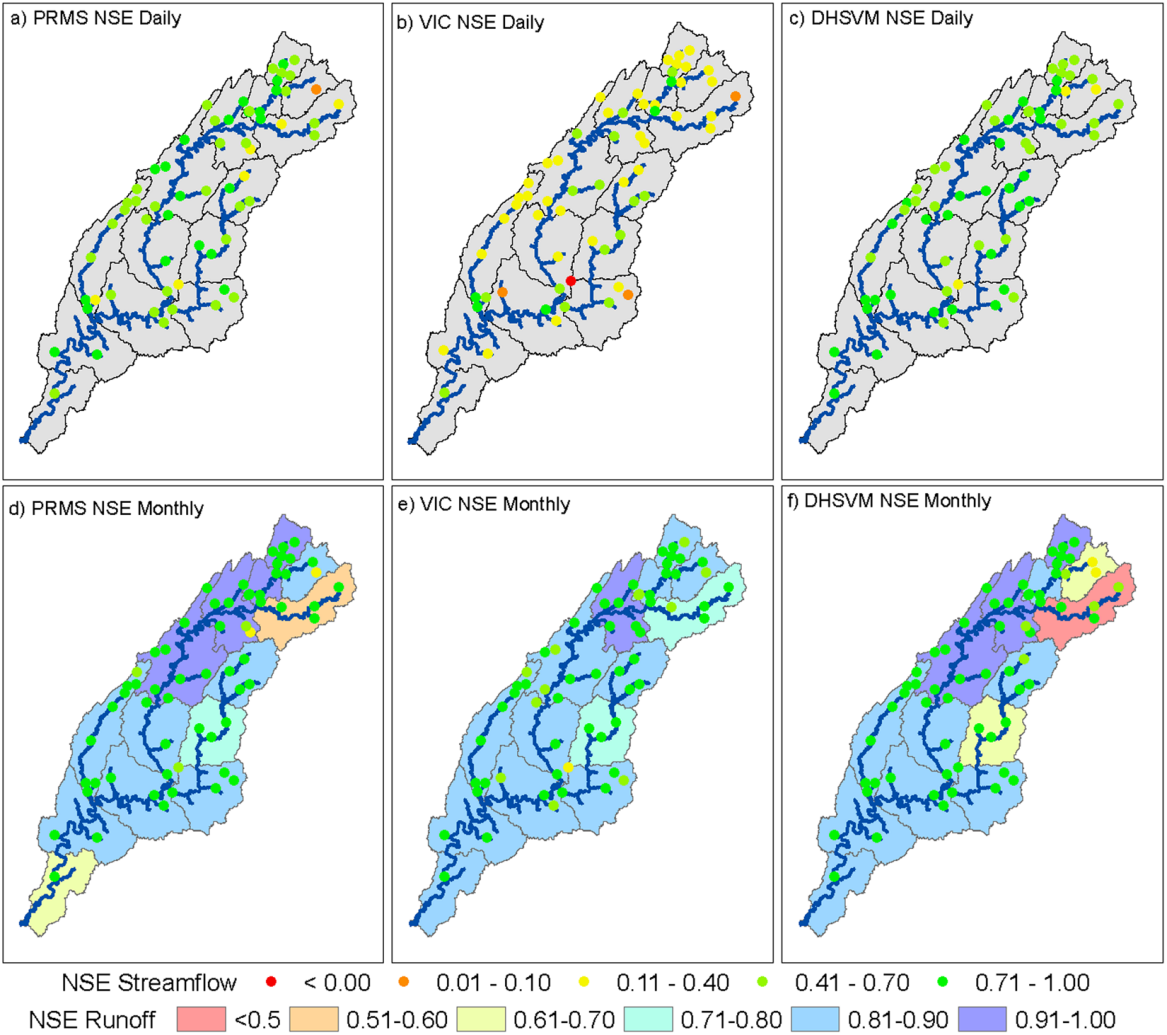
Historic model performance at daily and monthly scales for each of the three hydrologic models. The HUC08s and USGS gauge locations are color-coded based on NSE values. The simulated streamflow values are compared with corresponding observed historic USGS streamflow, while the simulated runoff was compared with USGS WaterWatch runoff as a benchmark for the period of 1981–2012. The figure was created using ESRI ArcMap (https://desktop.arcgis.com/en/arcmap/) Version 10.6.1.9270.

The time series of simulated monthly runoff was compared with the observed runoff from USGS WaterWatch for each HUC08. The key statistics including aggregate annual runoff, Nash–Sutcliffe Efficiency (NSE) are presented (Fig. 2, and SI Table S1). The NSE values at the monthly time step ranged between 0.43–0.93 for VIC, 0.59–0.92 for PRMS, and 0.74–0.91 for DHSVM. For all hydrologic models, 11 out of 14 HUC08s exhibited NSE values greater than 0.8, demonstrating skillful hydrologic models. Since the USGS WaterWatch data include gauges under influence of regulation, basins with large reservoir storage could show a potential bias.

The next comparison included evaluation of daily streamflow for 62 USGS gauges spread over the entire ACT River Basin covering every HUC08 (Fig. 2). Additional information about key statistics such as NSE at both daily and monthly time steps for each hydrologic model and for each gauge location is presented in SI Table S2. At the monthly scale, all three models demonstrated the ability to recreate historic USGS streamflow. For instance, the monthly NSE values were greater than 0.7 for roughly 79% of USGS gauges for VIC, 92% for PRMS, and 89% of USGS gauges for DHSVM (Fig. 2d–f, and SI Table S2). However, the VIC model had a lower performance at the daily scale compared with DHSVM and PRMS (Fig. 2a–c and SI Table S2). The USGS gauge closest to the outlet of the ACT River Basin demonstrated a similar level of performance for all three models with monthly NSE values of 0.89 for PRMS, 0.90 for VIC, and 0.91 for DHSVM. Note that the current hydrologic model setup for each model does not incorporate the effects of reservoirs on the streamflow; therefore, model performance was affected for the USGS gauges located immediately downstream of large reservoirs. The effect of regulation from reservoirs on hydrograph response tended to dissipate for the gauges further downstream. Overall, the results suggest a satisfactory performance of hydrologic models in the historic period.

### Future hydroclimate projections

#### Precipitation

The ΔP averaged over the ACT River Basin is projected to increase across all the seasons (SI Fig. S3). The multi-model mean precipitation exhibited an increased by 2.3%, 4.4%, 1.9%, and 3.5% during winter, spring, summer, and fall, respectively. During winter and spring seasons, the increase was generally observed across the entire basin with minor spatial variability. However, summer and fall exerted greater spatial variabilities in ΔP, with a slight decrease projected over the northeastern part of the ACT River Basin.

#### Runoff

**Figure 3:**
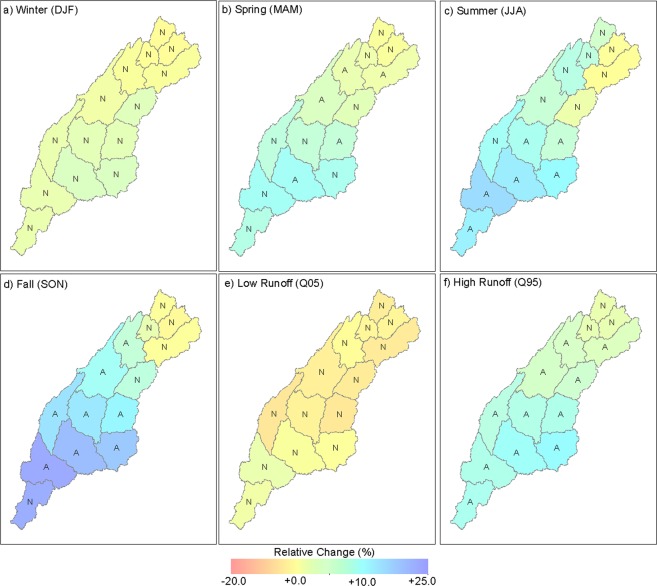
Projected changes in average monthly seasonal runoff (**a**–**d**), low runoff (**e**), and high runoff (**f**) over the ACT River Basin aggregated at HUC08 levels. The HUC08s with more than two-thirds of ensemble members indicating the same sign of change are labeled as “A” or otherwise “N.” The figure was created using ESRI ArcMap (https://desktop.arcgis.com/en/arcmap/) Version 10.6.1.9270.

Figure 3a–d and Table 1 present projected mean seasonal change in ΔR using all 33 ensemble members summarized at the HUC08 level for the ACT River Basin. The ΔR aggregated for the ACT River Basin (Table 1) suggests that average runoff is likely to increase by 2.3%, 5.5%, 8.0%, and 12.0% in winter, spring, summer and fall, respectively. The spatial distribution of ΔR indicates that the lower half of the basin may potentially observe a larger increase in runoff along the Alabama River compared with the upstream tributaries of the Coosa River (HUC08s such as 03150102 and 03150104). Furthermore, the spatial variability in ΔR is largest in fall and summer compared with winter and spring. The spatial patterns and seasonal changes in ΔR are generally consistent with ΔP. Changes in low runoff indicate a projected decrease by −1.74%, while high runoff is projected to increase by 6.6% averaged across the ACT River Basin. Low runoff is projected to change within a range of −6.6% to +1.6% for roughly 72% of the HUC08s (Fig. 3e). Similarly, high runoff is projected to increase for all HUC08s within a range of +1.5% to +10.8% (Fig. 3f).

**Table 1:** Summary statistics for mean ensemble percent change in runoff observed under climate change summarized by HUC08s in the ACT River Basin.

HUC08	Change in Runoff (%)					
	Winter	Spring	Summer	Fall	Q05	Q95
03150101	−0.34	0.49	5.15	0.47	0.72	1.46
03150102	−0.32	0.40	0.13	−0.82	−3.25	2.24
03150103	0.01	1.84	6.86	2.60	−0.41	2.46
03150104	0.51	1.87	0.02	0.22	−3.12	3.89
03150105	0.64	2.43	7.70	6.36	1.68	3.70
03150106	1.50	3.93	6.10	9.14	−2.67	5.15
03150107	3.22	6.20	8.70	13.02	−2.49	6.71
03150108	3.60	3.38	2.12	7.16	−3.20	4.51
03150109	3.29	6.25	6.19	11.41	−6.56	8.34
03150110	4.13	8.33	10.56	17.73	−3.05	10.78
03150201	4.06	9.16	12.35	19.42	−0.08	9.77
03150202	2.28	6.80	9.28	13.89	−3.70	7.30
03150203	2.55	8.99	15.07	22.39	1.34	9.16
03150204	2.26	7.72	12.10	21.85	1.31	7.61
**ACT River Basin**	**2.28**	**5.53**	**7.97**	**11.99**	**−1.74**	**6.60**

The robustness of hydrologic projections is evaluated for each variable and for each HUC08 in Fig. 3. The HUC08s with more than two-thirds of ensemble members indicating a same sign of change are labeled as “A”; otherwise, they are labeled “N.” These results suggest that roughly 35%, 42%, 57%, and 79% of HUC08s indicate an agreement during spring, summer, fall, and Q95, while no agreement was observed during winter and Q05.

#### Streamflow

**Figure 4:**
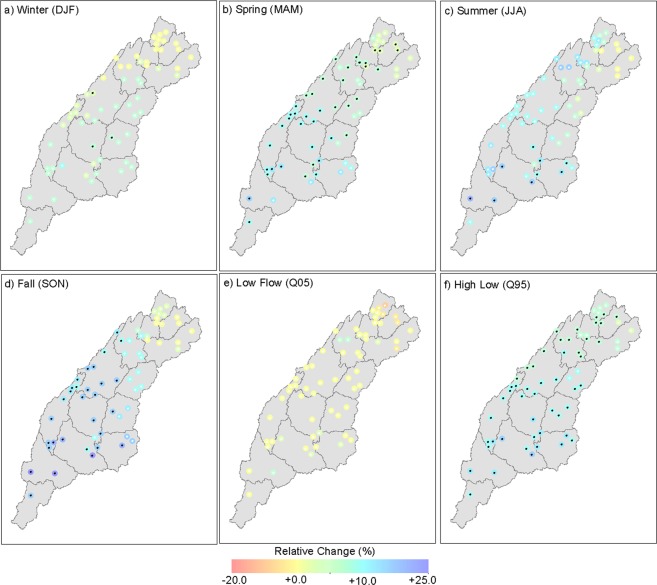
Projected changes in average monthly seasonal streamflow (**a**–**d**), low flow (**e**), and high flow (**f**) over the ACT River Basin for each USGS gauge location. The gauge locations with more than two-thirds of ensemble members indicating the same sign of change are stippled in black. The figure was created using ESRI ArcMap (https://desktop.arcgis.com/en/arcmap/) Version 10.6.1.9270.

Next, future changes in streamflow for 62 selected USGS gauges in the ACT River Basin were evaluated. Although runoff provides a good sense of overall water distribution in the basin, evaluating streamflow can directly indicate water availability in the channels. Therefore, the response of streamflow, particularly high and low flows, under climate change is of interest to water managers. Figure 4a–d presents projected changes in ΔQ for each of the USGS gauge locations with detailed statistics presented in SI Table S3. The projected change ranges in seasonal streamflow for 62 USGS gauges are as follows: winter (−1.2% to +5.2%), spring (+0.9% to +10%), summer (−2.3% to +18.1%), and fall (−2.2% to +23.4%). Maximum changes are projected in the months of summer and fall with up to a 23% increase in the streamflow in future. The spatial distribution of changes in streamflow indicates a larger increase in the lower half of the basin, whereas a moderate change is observed in the upper half of the basin. While the spatial pattern of ΔQ is consistent with runoff changes, the magnitudes of projected changes in ΔQ are larger than ΔR.

Figure 4e,f show projected percent changes in streamflow extremes ΔQ05 and ΔQ95, respectively, for each USGS gauge location. The ΔQ95 is projected to increase between 1.8% and 11.1% across all gauges. In general, ΔQ95 will increase by approximately 4.0% averaged across gauges located in the Coosa River (HUC08s 03150101 through 03150107), and approximately 7.2% averaged across gauges in the Tallapoosa River and Alabama River (SI Table S3). A similar evaluation for ΔQ05 (Fig. 4e, SI Table S3) revealed that ΔQ05 is projected to decrease across 84% of the gauges along with greater spatial heterogeneity across the ACT River Basin. Most gauges located in the upstream HUC08s (03150101, 03150102, and 03150104) exhibited an average projected decrease of 4.7% in low flows with a maximum change of approximately −19.7%. The rest of the gauges in the lower half of the ACT River Basin exhibited an average projected decrease of roughly −1.6% in the low flows.

A comparison of the hydrologic projections generated by different hydrologic models for gauge A0096 (USGS gauge closest to the outlet of the ACT River Basin) revealed that PRMS, VIC, and DHSVM suggest a mean change (ensemble range) in projected streamflow by +3.2% (−23.3% to +16.0%), +6.4% (−19.0% to +21.1%), and +6.0% (−13.1% to +21.1%), respectively. In general, PRMS resulted in a relatively lower change in mean streamflow signal response compared with VIC and DHSVM. However, the ensemble range was much larger and was comparable among the hydrologic model, suggesting that despite the differences in the model structures, resolution, calibration, and validation, these three hydrologic models provide similar insights in hydrologic projections.

### Role of climate versus hydrological models and uncertainty evaluation

**Figure 5:**
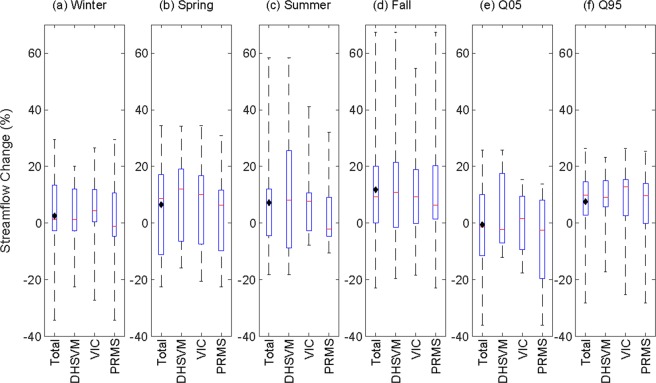
Distribution of percent change in mean, high, and low streamflow for a selected USGS gauge close to the outlet of the ACT River Basin. In each panel, “Total” represents distributions obtained from all 33 projections, while DHSVM and VIC represent distributions obtained from the respective choice of hydrologic model. The multi-model mean for total ensemble is shown as a black diamond.

As discussed in the Introduction, uncertainties are evident in future hydroclimate projections derived through the hierarchical modeling chain introduced by various factors. While ensemble mean values of projections can be beneficial, ranges in ensemble values for future projections can also serve as important information for water resource management. As indicated in the previous section, the mean hydrologic response from each hydrologic model captures similar information in streamflow change; this analysis further provides a breakdown for seasonal, high, low flows presented for gauge A0096, for example. The range associated with change in hydroclimate response, in addition to mean hydrologic signal, is also presented in Fig. 5 for different streamflow variables (ΔQ at seasonal scale, ΔQ05, and ΔQ95) arising from 33 sets of hydroclimate projections. Each subfigure (Fig. 5a–f), provides an ensemble range of ΔQ arising from individual hydrologic models (PRMS, VIC, and DHSVM) and compared with “Total” (ensemble range from 33 members). In each subfigure, the distribution spreads of relative change in flow obtained by individual hydrologic models are very similar to each other. In other words, the distribution is not significantly different from one hydrologic model to the next. This indicates that the choice of hydrologic model is not as significant as selecting a climate model because the total spread is largely driven by uncertainties associated with precipitation arising from different climate models. In general, a similar trend was observed in most of the remaining USGS gauge locations. Results for four additional upstream locations within ACT River Basin including A0019, A0063, A0078 and A0092, which are representative of tributaries to Alabama River, and corresponds to Cahaba River, Coosa River, Tallapoosa River and Oostanaula River respectively are presented in SI Fig. S5. However, higher uncertainty was observed in the simulation of ΔQ during summer, fall, and ΔQ05 for a few gauges located in the northeastern part of the ACT River Basin.

**Figure 6:**
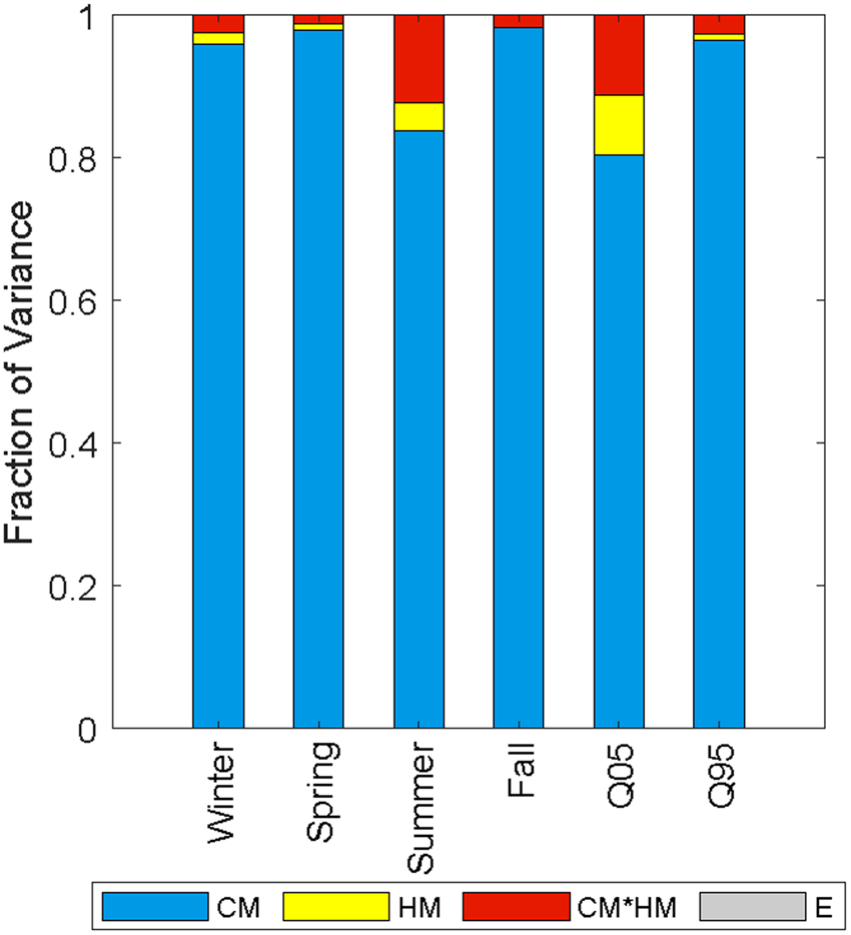
Relative contribution of different sources of uncertainty to total variance for each hydrologic indices. The CM, HM, CM × HM, and E represent variance caused by climate models, hydrologic models, interaction of climate and hydrologic models, and error.

ANOVA also provided the relative contribution of uncertainties arising from climate models and hydrologic models to total ensemble uncertainty. The variance decomposition suggested that CM was the dominant source of variability, accounting for over 80% of total variance for all six variables (Fig. 6). The second largest source of variability arose from the interaction of climate and hydrologic models (CM × HM). The contribution of the HM was relatively low compared with other factors, while the residual error was almost negligible in all cases. An increase in relative contribution from HM was observed for summer flow and low flow conditions, indicating a relatively stronger influence of the choice of hydrologic models in conditions where baseflow constitutes a larger portion of streamflow. However, since all thee hydrologic models used in this study were robustly calibrated, the lesser influence of the HM was expected in SEUS, whereas the opposite may be expected for drier or snow-dominated regions.

### Discussion and potential implications

The projected changes in seasonal hydrology demonstrate that the ACT River Basin, in general, is expected to experience an increase in total runoff and streamflow in the future, which could be attributed to an overall increase in the seasonal precipitation over the region. However, the magnitude of the increase in runoff is not linearly proportional to the increase in precipitation. A small increase of 1.9% in precipitation potentially causes an increase of 8.0% in runoff during summer. Similar behavior was also exhibited during the fall season. This result could be attributed to the high hydrologic sensitivity of runoff and indicates that even a small increase in precipitation could yield a significant increase in runoff response^45^. Moreover, increases in high-intensity storm events can trigger high runoff response signals despite relatively marginal increases in total seasonal precipitation. This finding suggests that the summer and fall seasons in the SEUS could observe an increase in precipitation intensity. This explanation corroborates the results of other studies indicating ongoing intensification of summer^46,47^ and fall^48^ precipitation in the SEUS based on historic observed datasets and reanalysis datasets. Such behavior is exhibited by the regionally downscaled precipitation extremes under future climate conditions over ACT river basin (SI Fig. S4) The projected changes in high flows further indicate that climate change will likely affect the frequency and magnitude of high-flow events consistently throughout the ACT River Basin; these effects may be further exacerbated if urbanization and deforestation occur under future conditions in the region (not accounted for explicitly in hydrologic models). The changes are more prominent for the lower half of the basin, including the gauges located around Martin Dam, Jordan Lake, Robert F. Henry Lock and Dam, Millers Ferry, and Claiborne Reservoir. The changes in low flows are more prominent in the northwestern parts of the ACT River Basin. Projected increases in high flows and ubiquitous decreases in low flows across the majority of gauges in the ACT River Basin suggest an intensification of extremes in the hydrologic cycle in the region under future climatic conditions.

The projected seasonal and high/low streamflow changes provide valuable information to water resource managers and other reservoir operators in the region. Despite only moderate increases projected for high flows, such information is still beneficial for infrastructure design and safety. Likewise, projected decreases in low flows during summer and fall for the upper half of the ACT River Basin could influence reservoir operations, especially during periods when reservoir operations balance competing demands such as water supply, hydropower, minimum environmental and recreational flow, and others.

Based on the uncertainty quantification for the ACT River Basin, the choice of GCM is the most important factor when designing the hierarchical modeling framework for impact studies. The choice of hydrologic models plays an insignificant role in uncertainty of hydrologic regimes in the region relative to the uncertainties arising from the climate projections. A complex and computationally intensive hydrologic model such as DHSVM provides similar insights in hydrologic projections compared to VIC and PRMS in this region, suggesting that water managers and other stakeholders can place greater emphasis on the selection of climate models for future hydroclimate study designs. It is important to note that while we use the hydrologic models with varying spatial resolution in this study, the effect of resolution is inseparable from the other factors (such as model parameterization) in the current study design.

Internal variability of GCMs is not explicitly calculated, as this commonly requires generating multiple simulations for a given GCM using different initial conditions but similar external forcings^49^. Since the meteorological forcings for this study were only limited by one run per GCM, the internal variability is therefore integral in determining GCM uncertainty. Nevertheless, the findings of this study align with other studies that focused on quantifying the major sources of uncertainty over various regions^8–10,23,44^.

## Summary and Conclusions

Evaluations of future water resources under a changing climate require reliable hydroclimate projections. These projections are often generated by driving calibrated hydrologic models using meteorological outputs from GCMs. In this study, a hydro-meteorological framework of process-based models was developed. Using a combination of 11 dynamically downscaled GCMs and 3 calibrated hydrologic models, 33 hydrologic projections over the ACT River Basin were produced. The future projections were generated under the RCP8.5 emission scenario for a 40-year period of 2011–2050, which were compared with baseline simulations (1966–2005). The high-resolution simulated hydrologic outputs variables were analyzed and sources of uncertainties arising from climate models and hydrologic models were quantified.

Overall, models are reasonably able to simulate baseline hydroclimates comparable to the observations. The future projections demonstrated an increase in multi-model mean seasonal precipitation during all seasons by 1.9% to 4.4% relative to baseline. The runoff signal exhibited a similar behavior; however, the changes in runoff were not linearly proportional to the increase in precipitation. For instance, the summer season observed an 8% increase in runoff while precipitation increased only by 2%. This finding indicates future intensification of summer rainfall consistent with existing trends documented in other studies, as discussed previously. The consistent increase projected in high flow further suggests an increasing trend of high-intensity rainfall across the ACT River Basin, whereas the projected low flow exhibits a decreasing trend for most gauge locations, indicating potential slight intensification of the hydrological cycle in the region. The increased magnitudes of high-flow events could put additional stress on major reservoirs with the primary goal of flood control in the ACT River Basin. On the other hand, the decreased low flow magnitudes could make reservoirs more vulnerable when they encounter competing water demands.

The analysis of changes in seasonal and extreme flows close to the outlet of the ACT River Basin showed a large distribution spread, which was consistent across most gauges. A quantification of sources of uncertainties using ANOVA revealed that climate models are the dominant source of uncertainties in the region. These results were consistent across all measures of streamflow. The results suggest that different hydrologic models do not yield different insights about hydroclimate projection at the watershed scale, thereby suggesting that if resources are limited, water managers can use a relatively coarser/simpler hydrologic model to effectively capture the hydrologic projections. Although this study considered two sources of uncertainties, other sources may be incorporated in the future. A more comprehensive analysis would incorporate additional sources of uncertainties by including other emission scenarios, climate models, downscaling approaches, sets of hydrologic parameters, and future land use cover. Despite the limitations, this study can set a path forward for applications of the proposed framework to many aspects of water resources, including investigation of future flood risks, water supply, reservoir operations, and hydropower production in the ACT River Basin.

## Supplementary information

Supplementary Information.

## Data Availability

The hydrologic models used in the study are open source and can be obtained from the following links: • PRMS: https://www.usgs.gov/software/precipitation-runoff-modeling-system-prms • VIC: https://vic.readthedocs.io/en/master/Development/ReleaseNotes/#vic-412-and-earlier • DHSVM: https://github.com/pnnl/DHSVM-PNNL The hydrologic projections generated in the study, model setup details, or any other pertinent information can be made available by the corresponding author upon request.
