# Supplementary material for: Multi-model Hydroclimate Projections for the Alabama-Coosa-Tallapoosa River Basin in the Southeastern United States
Source: Sci Rep. 2020 Feb 18;10:2870. doi: 10.1038/s41598-020-59806-6 (PMC7028922; doi:10.1038/s41598-020-59806-6)
Supplement: Supplementary file 1 — Supplementary Information. [file 41598_2020_59806_MOESM1_ESM.docx]

# Multi-model Hydroclimate Projections for the Alabama-Coosa-Tallapoosa River Basin in the Southeastern United States

Sudershan Gangrade,^1,2,3^ Shih-Chieh Kao,^1,2,3,*^ and Ryan A. McManamay ^4^

^1^ The Bredesen Center, University of Tennessee, Knoxville, TN 37996, USA

^2^ Climate Change Science Institute, Oak Ridge National Laboratory, Oak Ridge, TN 37831, USA

^3^ Environmental Sciences Division, Oak Ridge National Laboratory, Oak Ridge, TN 37831, USA

^4^ Department of Environmental Science, Baylor University, Waco, TX 76706, USA

*Corresponding Author

Shih-Chieh Kao, PhD

P.O. Box 2008, MS-6038

Oak Ridge, TN 37831-6038, USA

Telephone: 865-576-1259

Email Address: [kaos@ornl.gov](mailto:kaos@ornl.gov)

# Supplementary Information:

## Description of Regional Climate Modeling and Downscaling

Regional climate modeling and downscaling involved a hybrid downscaling approach. The GCM outputs were first dynamically downscaled to 18 km resolution using RegCM4 model^1^ and then statistically bias corrected to 1/24° (~4 km) resolution utilizing a quantile mapping approach^2,3^. It was conducted at the monthly scale and used the 1/24° (~4 km) resolution 1966–2005 monthly precipitation and temperature from the Parameter-elevation Regressions on Independent Slopes Model (PRISM) dataset as observation. The monthly correction values were then evenly distributed to daily time series of precipitation (using ratio adjustment) and temperature (using degree adjustment). More details are listed in the Ashfaq et al. (2016) and Naz et al. (2016). A further evaluation of RegCM4 precipitation outputs compared against PRISM over ACT river basin is presented in figures S1 through S4.

## Description of Hydrologic Models

#### Precipitation Runoff Modeling System

Precipitation Runoff Modeling System (PRMS) is a deterministic, process-based model, distributed hydrologic model developed by the United States Geological Survey ^4^. PRMS has a modular framework to enable use of alternative algorithms to simulate several hydrologic processes such as precipitation, evapotranspiration, runoff, infiltration, groundwater etc. ^5^. PRMS has been widely utilized to study climate change impacts at watershed scale ^6,7^. In this study, PRMS is implemented using NHM-PRMS ^8^ which utilizes Geospatial Fabric ^9^ for spatial discretization to obtain hydrologic response units (HRUs) and stream segments, while the initial parameters were obtained through National Hydrologic Model Parameter Database ^10^. PRMS was setup for a period of 1980-2012 and first year was used a spinup. Six sensitive parameters to runoff and streamflow response were identified from literature ^11^ to calibrate PRMS using particle swarm optimization algorithm. The calibration was first carried out by maximizing Nash Sutcliffe Efficiency (NSE) values individually at HUC08 level by comparing the simulated runoff with observed monthly runoff obtained from USGS WaterWatch runoff dataset at monthly scale. Finally, the “K_coef” parameter was adjusted for all the channel segments to maximize daily NSE values for USGS gagues within the HUC08 progressively starting from upstream to downstream.

#### Variable Infiltration Capacity Model (VIC)

VIC is a semi-distributed, grid-based, macroscale hydrologic model which solves energy and water balance equations using physical process-based equation (including hydrologic process such as evaporation, runoff, baseflow, energy fluxes etc.) within grid cell. The model utilizes a variable infiltration capacity curve to determine the infiltration and surface runoff process, while empirical Arno curve is used to generate base flow. VIC allows to represent sub-grid variability by accounting for topography, precipitation and vegetation ^12^. The grid cells do not interact with each other during the simulation, therefore, the streamflow estimates are produced by routing the surface runoff and baseflow from each grid cell to desired location through the river network based on a linear reservoir model ^13,14^.

In this study, the VIC hydrologic model was setup at 1/24° (~4 km) grid resolution with a three-hourly time step using five elevation bands for each grid cell. The model was calibrated for each HUC08 by comparing the simulated runoff with observed monthly runoff obtained from USGS WaterWatch runoff dataset ^15^. The WaterWatch data provides aggregated monthly runoff derived from USGS National Water Information System gauges. Additional model setup details are available from Naz et al. (2016) and Oubeidillah et al. (2014) ^16,17^.

#### Distributed Hydrology Soil Vegetation Model

Distributed Hydrology-Soil-Vegetation Model (DHSVM) is a physical process based distributed hydrologic model typically implemented at 30-200 m resolution ^18^. DHSVM is primarily a saturation excess model ^19^ which solves energy balance and mass (water) balance equations at each grid cell. DHSVM utilizes spatially distributed parameters including topography, soil, soil depths, and vegetation type where each grid cell represents one soil type and one vegetation type. The input meteorological data comprises mainly of precipitation, incoming shortwave and longwave radiation, relative humidity, air temperature and wind speed. It captures the hydrological processes such as evapotranspiration, snowmelt, canopy snow interception and release, unsaturated soil moisture, saturated subsurface flow, overland flow and channel flow. A detailed description of DHSVM and physical process-based equations can be found in ^20-22^.

A calibrated DHSVM setup utilized in this study is obtained from Gangrade et al.

(2018) ^23^. The model is implemented at a 90-meter spatial resolution at sub-daily temporal scale (3-hourly). The calibration and validation of DHSVM was performed individually at sub-basin level employing 74 USGS gauges. Additional details about the calibration, validation and model setup are available from Gangrade et al. (2018) ^23^.

## Figures and Tables


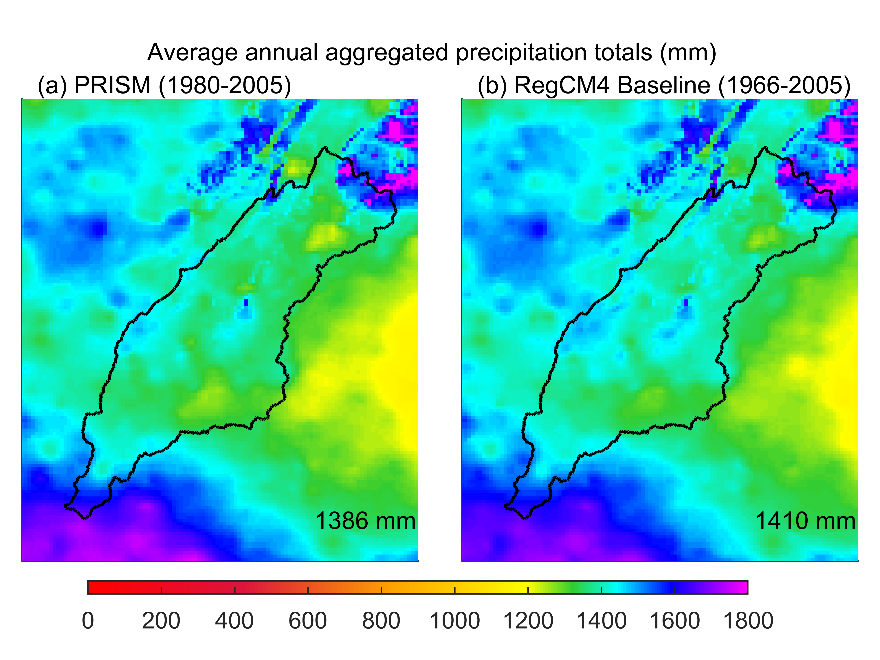


## Figure S1. Annual scale comparison – Evaluation of average annual aggregated precipitation totals for observation (1980–2005 PRISM) and multi-model mean RegCM4 during the baseline period (1966–2005). The average precipitation received by ACT River Basin is listed on the lower right corner at each panel. The figure was created using MathWorks MATLAB (https://www.mathworks.com/) R2019a.


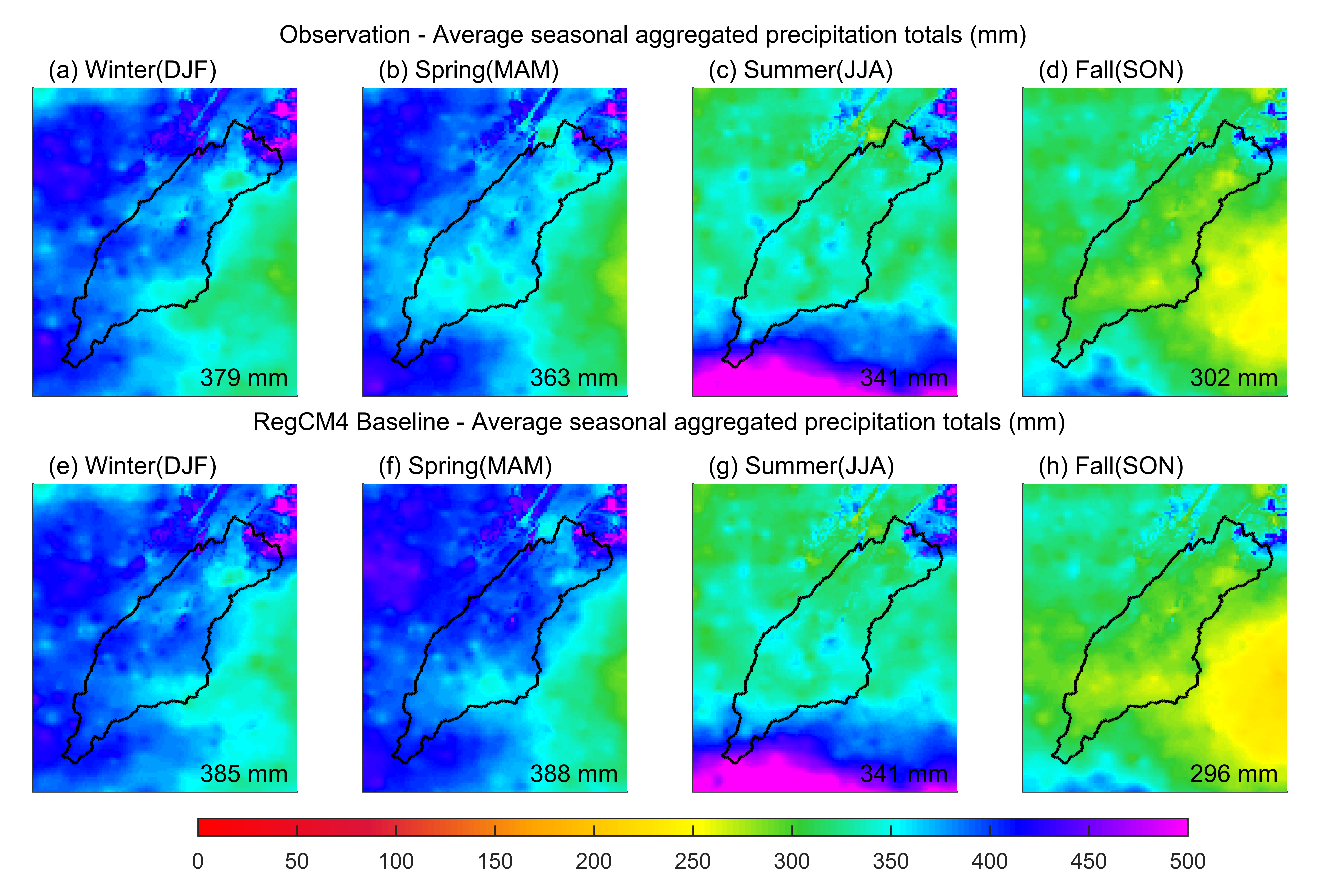


## Figure S2. Seasonal scale comparison – Evaluation of average seasonal aggregated precipitation totals for observation (1980–2005 PRISM) and multi-model mean RegCM4 during the baseline period (1966–2005). The average precipitation received by ACT River Basin is listed on the lower right corner at each panel. The figure was created using MathWorks MATLAB (https://www.mathworks.com/) R2019a.

## Figure S3. Projected percentage changes in average seasonal precipitation over ACT from baseline period (1966–2005) to future period (2011–2050). The changes are calculated as future minus baseline with respect to baseline. The figure was created using MathWorks MATLAB (https://www.mathworks.com/) R2019a.


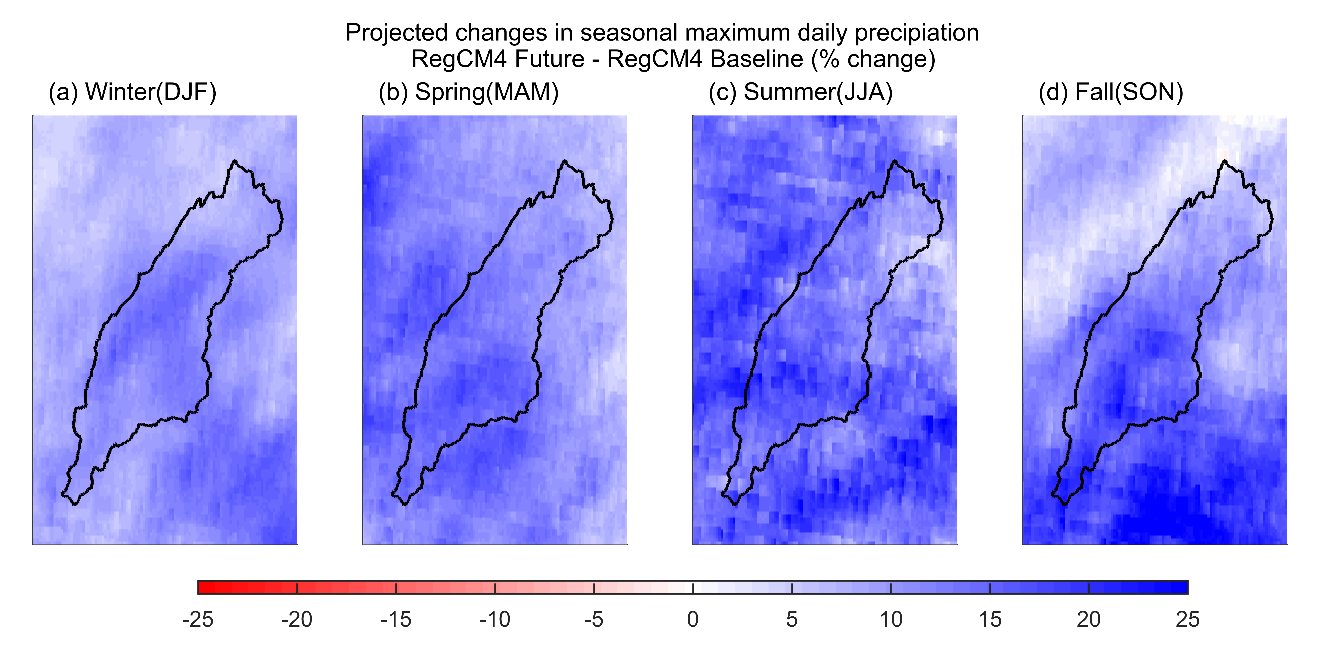


## Figure S4. Projected percentage changes in average seasonal maximum daily precipitation over ACT from baseline period (1966–2005) to future period (2011–2050). The changes are calculated as future minus baseline with respect to baseline. The figure was created using MathWorks MATLAB (https://www.mathworks.com/) R2019a.


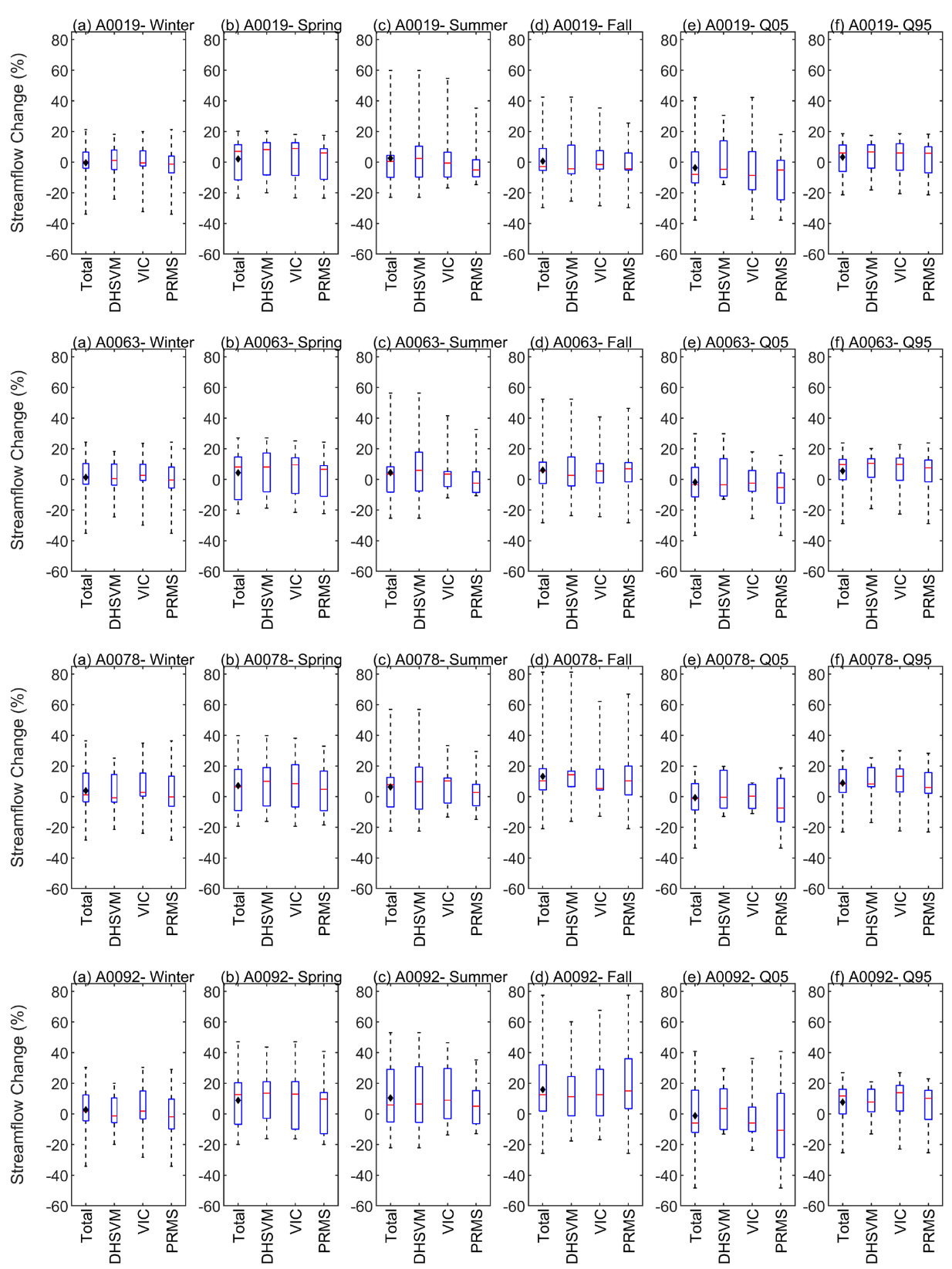


## Figure S5. Distribution of percent change in mean, high, and low streamflow four upstream locations within ACT river basin. Locations A0019, A0063, A0078 and A0092 corresponds to Cahaba River, Coosa River, Tallapoosa River and Oostanaula River respectively.

## Table S1. Summary Statistics for Hydrologic Model Performance by HUC08s in ACT River Basin

| HUC08 | Drainage Area  (sq. km) | Runoff (mm/yr) | | | | PBIAS (%) | | | NSE (monthly) | | |
| --- | --- | --- | --- | --- | --- | --- | --- | --- | --- | --- | --- |
|  |  | Water  Watch | VIC | DHSVM | PRMS | VIC | DHSVM | PRMS | VIC | DHSVM | PRMS |
| 03150101 | 1883 | 572.3 | 525.1 | 560.0 | 555.2 | -8.3 | -2.2 | -3.0 | 0.91 | 0.87 | 0.92 |
| 03150102 | 2232 | 550.5 | 527.2 | 524.6 | 584.6 | -4.2 | -4.7 | 6.2 | 0.61 | 0.86 | 0.82 |
| 03150103 | 1453 | 527.5 | 465.2 | 571.8 | 512.8 | -11.8 | 8.4 | -2.8 | 0.88 | 0.85 | 0.92 |
| 03150104 | 4821 | 483.5 | 492.1 | 475.4 | 541.7 | 1.8 | -1.7 | 12.0 | 0.43 | 0.74 | 0.59 |
| 03150105 | 4142 | 565.5 | 549.3 | 579.6 | 526.2 | -2.9 | 2.5 | -7.0 | 0.93 | 0.91 | 0.92 |
| 03150106 | 6692 | 518.8 | 503.4 | 492.8 | 488.5 | -3.0 | -5.0 | -5.8 | 0.93 | 0.90 | 0.91 |
| 03150107 | 5083 | 500.8 | 538.1 | 443.0 | 492.6 | 7.4 | -11.5 | -1.6 | 0.84 | 0.87 | 0.87 |
| 03150108 | 3612 | 474.0 | 501.0 | 460.1 | 452.6 | 5.7 | -2.9 | -4.5 | 0.83 | 0.85 | 0.88 |
| 03150109 | 4118 | 460.8 | 418.2 | 354.1 | 500.2 | -9.3 | -23.1 | 8.6 | 0.69 | 0.79 | 0.71 |
| 03150110 | 4411 | 411.4 | 423.4 | 387.4 | 436.1 | 2.9 | -5.8 | 6.0 | 0.82 | 0.85 | 0.82 |
| 03150201 | 6193 | 433.5 | 426.3 | 456.1 | 390.2 | -1.7 | 5.2 | -10.0 | 0.89 | 0.85 | 0.88 |
| 03150202 | 4724 | 506.3 | 473.0 | 538.2 | 473.8 | -6.6 | 6.3 | -6.4 | 0.90 | 0.90 | 0.90 |
| 03150203 | 5775 | 442.8 | 426.2 | 437.0 | 421.4 | -3.7 | -1.3 | -4.8 | 0.82 | 0.88 | 0.88 |
| 03150204 | 3758 | 493.2 | 430.8 | 443.5 | 514.6 | -12.7 | -10.1 | 4.3 | 0.81 | 0.80 | 0.69 |

## Table S2. Summary Statistics for Hydrologic Model Performance by HUC08s in ACT River Basin

| UniqueID | USGSID | Longitude | Latitude | Drainage Area (sq. km) | Data Availability | | NSE monthly | | | NSE daily | | |
| --- | --- | --- | --- | --- | --- | --- | --- | --- | --- | --- | --- | --- |
|  |  |  |  |  |  |  | VIC | DHSVM | PRMS | VIC | DHSVM | PRMS |
| A0001 | USGS02380500 | -84.51 | 34.68 | 611 | 1938 | 2013 | 0.85 | 0.37 | 0.75 | 0.29 | 0.53 | -0.14 |
| A0002 | USGS02381600 | -84.47 | 34.57 | 26 | 1974 | 2013 | 0.65 | 0.27 | 0.20 | 0.15 | 0.38 | 0.06 |
| A0007 | USGS02383500 | -84.83 | 34.56 | 2,152 | 1938 | 2013 | 0.89 | 0.63 | 0.87 | 0.31 | 0.38 | 0.46 |
| A0008 | USGS02384500 | -84.85 | 34.83 | 653 | 1981 | 2013 | 0.78 | 0.84 | 0.92 | 0.28 | 0.70 | 0.79 |
| A0009 | USGS02384540 | -84.72 | 34.87 | 21 | 1985 | 2013 | 0.51 | 0.76 | 0.82 | 0.12 | 0.59 | 0.63 |
| A0010 | USGS02385170 | -84.88 | 34.74 | 456 | 2005 | 2013 | 0.71 | 0.81 | 0.87 | 0.28 | 0.64 | 0.70 |
| A0011 | USGS02385500 | -84.97 | 34.79 | 104 | 1943 | 2013 | 0.73 | 0.76 | 0.90 | 0.23 | 0.61 | 0.49 |
| A0012 | USGS02385800 | -84.77 | 34.72 | 166 | 1960 | 2013 | 0.76 | 0.82 | 0.79 | 0.15 | 0.62 | 0.67 |
| A0013 | USGS02387000 | -84.93 | 34.67 | 1,779 | 1937 | 2013 | 0.85 | 0.91 | 0.92 | 0.53 | 0.81 | 0.83 |
| A0014 | USGS02387500 | -84.94 | 34.58 | 4,149 | 1900 | 2013 | 0.92 | 0.84 | 0.93 | 0.76 | 0.79 | 0.82 |
| A0016 | USGS02388300 | -85.27 | 34.37 | 38 | 1968 | 1989 | 0.54 | 0.90 | 0.89 | 0.25 | 0.64 | 0.73 |
| A0017 | USGS02388320 | -85.26 | 34.37 | 43 | 1982 | 2013 | 0.60 | 0.82 | 0.82 | 0.28 | 0.65 | 0.64 |
| A0018 | USGS02388350 | -85.14 | 34.36 | 580 | 2005 | 2013 | 0.73 | 0.91 | 0.85 | 0.38 | 0.76 | 0.75 |
| A0019 | USGS02388500 | -85.14 | 34.30 | 5,478 | 1939 | 2013 | 0.92 | 0.88 | 0.94 | 0.78 | 0.81 | 0.85 |
| A0023 | USGS02390000 | -84.21 | 34.43 | 231 | 1939 | 2013 | 0.88 | 0.46 | 0.76 | 0.05 | 0.50 | 0.33 |
| A0029 | USGS02392000 | -84.49 | 34.24 | 1,588 | 1900 | 2013 | 0.88 | 0.59 | 0.82 | 0.35 | 0.70 | 0.53 |
| A0031 | USGS02392780 | -84.50 | 34.12 | 360 | 2005 | 2013 | 0.71 | 0.74 | 0.82 | 0.20 | 0.63 | 0.51 |
| A0038 | USGS02395120 | -84.89 | 34.24 | 86 | 1980 | 2013 | 0.58 | 0.70 | 0.79 | 0.14 | 0.49 | 0.33 |
| A0042 | USGS02397410 | -85.26 | 34.00 | 169 | 1981 | 2013 | 0.82 | 0.78 | 0.12 | 0.11 | 0.66 | 0.23 |
| A0043 | USGS02397500 | -85.31 | 34.06 | 298 | 1942 | 2013 | 0.85 | 0.40 | 0.69 | 0.19 | 0.60 | 0.54 |
| A0044 | USGS02398000 | -85.34 | 34.47 | 497 | 1937 | 2013 | 0.88 | 0.84 | 0.92 | 0.27 | 0.80 | 0.80 |
| A0046 | USGS02398300 | -85.51 | 34.29 | 948 | 1959 | 2013 | 0.89 | 0.83 | 0.91 | 0.45 | 0.80 | 0.72 |
| A0047 | USGS02399200 | -85.68 | 34.29 | 515 | 1958 | 2013 | 0.73 | 0.85 | 0.72 | 0.24 | 0.65 | 0.65 |
| A0048 | USGS02400100 | -85.61 | 34.07 | 653 | 1962 | 2013 | 0.86 | 0.78 | 0.87 | 0.44 | 0.76 | 0.69 |
| A0049 | USGS02400680 | -85.77 | 34.44 | 143 | 2002 | 2013 | 0.77 | 0.78 | 0.88 | 0.12 | 0.68 | 0.63 |
| A0050 | USGS02401000 | -86.04 | 34.10 | 471 | 1943 | 2013 | 0.85 | 0.90 | 0.90 | 0.43 | 0.78 | 0.83 |
| A0051 | USGS02401370 | -86.38 | 33.81 | 117 | 1978 | 1995 | 0.72 | 0.84 | 0.88 | 0.21 | 0.58 | 0.72 |

Table S2. Continued.

| UniqueID | USGSID | Longitude | Latitude | Drainage Area (sq. km) | Data Availability | | NSE monthly | | | NSE daily | | |
| --- | --- | --- | --- | --- | --- | --- | --- | --- | --- | --- | --- | --- |
|  |  |  |  |  |  |  | VIC | DHSVM | PRMS | VIC | DHSVM | PRMS |
| A0052 | USGS02401390 | -86.26 | 33.84 | 365 | 1965 | 2013 | 0.68 | 0.82 | 0.78 | 0.28 | 0.66 | 0.71 |
| A0055 | USGS02403310 | -85.79 | 33.60 | 495 | 2002 | 2011 | 0.84 | 0.90 | 0.88 | 0.53 | 0.84 | 0.70 |
| A0056 | USGS02404400 | -86.10 | 33.55 | 1,246 | 1960 | 2013 | 0.89 | 0.87 | 0.89 | 0.52 | 0.79 | 0.74 |
| A0057 | USGS02405500 | -86.39 | 33.45 | 500 | 1951 | 2013 | 0.59 | 0.83 | 0.73 | 0.31 | 0.66 | 0.67 |
| A0058 | USGS02406500 | -86.23 | 33.36 | 388 | 1900 | 2013 | 0.86 | 0.80 | 0.87 | 0.24 | 0.73 | 0.75 |
| A0060 | USGS02407514 | -86.50 | 33.32 | 368 | 2005 | 2013 | 0.67 | 0.88 | 0.82 | 0.39 | 0.76 | 0.68 |
| A0061 | USGS02408540 | -86.27 | 32.92 | 681 | 1980 | 2013 | 0.89 | 0.89 | 0.91 | 0.21 | 0.68 | 0.72 |
| A0062 | USGS02410000 | -86.13 | 32.68 | 13 | 1953 | 1987 | 0.35 | 0.77 | 0.49 | -0.08 | 0.39 | 0.25 |
| A0063 | USGS02411000 | -86.25 | 32.61 | 25,900 | 1912 | 2013 | 0.91 | 0.91 | 0.91 | 0.56 | 0.69 | 0.64 |
| A0065 | USGS02411930 | -85.34 | 33.74 | 704 | 1999 | 2013 | 0.73 | 0.68 | 0.77 | 0.21 | 0.71 | 0.31 |
| A0066 | USGS02412000 | -85.51 | 33.62 | 1,160 | 1952 | 2013 | 0.80 | 0.84 | 0.90 | 0.36 | 0.78 | 0.77 |
| A0068 | USGS02413210 | -85.28 | 33.49 | 635 | 2000 | 2013 | 0.75 | 0.80 | 0.87 | 0.46 | 0.72 | 0.50 |
| A0069 | USGS02413300 | -85.40 | 33.44 | 1,052 | 1975 | 2013 | 0.80 | 0.84 | 0.90 | 0.48 | 0.77 | 0.57 |
| A0070 | USGS02414500 | -85.56 | 33.12 | 4,338 | 1923 | 2013 | 0.84 | 0.82 | 0.86 | 0.49 | 0.65 | 0.65 |
| A0072 | USGS02414715 | -85.74 | 32.98 | 5,330 | 1985 | 2013 | 0.84 | 0.83 | 0.86 | 0.51 | 0.70 | 0.71 |
| A0073 | USGS02415000 | -85.88 | 33.07 | 492 | 1952 | 2013 | 0.88 | 0.80 | 0.86 | 0.18 | 0.67 | 0.75 |
| A0074 | USGS02418230 | -85.59 | 32.63 | 185 | 1999 | 2013 | 0.74 | 0.87 | 0.85 | 0.10 | 0.73 | 0.75 |
| A0076 | USGS02418760 | -85.48 | 32.55 | 119 | 2002 | 2013 | 0.55 | 0.73 | 0.71 | 0.06 | 0.55 | 0.57 |
| A0077 | USGS02419000 | -85.69 | 32.48 | 862 | 1939 | 2013 | 0.82 | 0.88 | 0.85 | 0.40 | 0.76 | 0.61 |
| A0078 | USGS02419890 | -86.20 | 32.44 | 12,033 | 1995 | 2013 | 0.84 | 0.82 | 0.83 | 0.66 | 0.70 | 0.62 |
| A0080 | USGS02420000 | -86.41 | 32.41 | 38,850 | 1927 | 2013 | 0.91 | 0.88 | 0.90 | 0.70 | 0.75 | 0.65 |
| A0081 | USGS02421000 | -86.30 | 32.31 | 751 | 1952 | 2013 | 0.66 | 0.85 | 0.81 | 0.19 | 0.62 | 0.65 |
| A0083 | USGS02422500 | -86.90 | 32.58 | 526 | 1938 | 2013 | 0.69 | 0.79 | 0.71 | 0.08 | 0.75 | 0.44 |
| A0084 | USGS02423130 | -86.60 | 33.62 | 51 | 1988 | 2013 | 0.65 | 0.78 | 0.68 | 0.12 | 0.52 | 0.52 |
| A0085 | USGS02423380 | -86.71 | 33.48 | 363 | 1980 | 2013 | 0.77 | 0.90 | 0.71 | 0.27 | 0.74 | 0.65 |
| A0086 | USGS02423400 | -86.61 | 33.50 | 63 | 1986 | 2013 | 0.76 | 0.79 | 0.80 | 0.18 | 0.53 | 0.62 |
| A0087 | USGS02423425 | -86.74 | 33.42 | 521 | 1975 | 2013 | 0.77 | 0.77 | 0.86 | 0.28 | 0.67 | 0.59 |

Table S2. Continued.

| UniqueID | USGSID | Longitude | Latitude | Drainage Area (sq. km) | Data Availability | NSE monthly | | | NSE daily | | |
| --- | --- | --- | --- | --- | --- | --- | --- | --- | --- | --- | --- |
|  |  |  |  |  |  | VIC | DHSVM | PRMS | VIC | DHSVM | PRMS |

| A0088 | USGS02423555 | -86.88 | 33.28 | 868 | 1995 | 2013 | 0.80 | 0.88 | 0.81 | 0.37 | 0.79 | 0.63 |
| --- | --- | --- | --- | --- | --- | --- | --- | --- | --- | --- | --- | --- |
| A0089 | USGS02424000 | -87.14 | 32.95 | 2,660 | 1901 | 2013 | 0.87 | 0.84 | 0.93 | 0.40 | 0.59 | 0.70 |
| A0090 | USGS02424590 | -87.20 | 32.53 | 3,833 | 1987 | 2011 | 0.90 | 0.89 | 0.92 | 0.73 | 0.75 | 0.82 |
| A0091 | USGS02424940 | -87.09 | 32.53 | 570 | 1975 | 1987 | 0.87 | 0.81 | 0.84 | 0.68 | 0.71 | 0.11 |
| A0092 | USGS02425000 | -87.18 | 32.44 | 4,574 | 1938 | 2013 | 0.91 | 0.92 | 0.93 | 0.76 | 0.76 | 0.81 |
| A0094 | USGS02427250 | -87.07 | 32.00 | 676 | 1989 | 2013 | 0.81 | 0.85 | 0.91 | 0.23 | 0.80 | 0.78 |
| A0095 | USGS02427700 | -87.56 | 32.02 | 253 | 1958 | 1996 | 0.76 | 0.86 | 0.90 | 0.17 | 0.77 | 0.79 |
| A0096 | USGS02428400 | -87.55 | 31.62 | 54,390 | 1975 | 2013 | 0.90 | 0.91 | 0.88 | 0.70 | 0.77 | 0.61 |

## Table S3. Summary statistics for percent change in seasonal streamflow (ΔQavg%), high flow (ΔQ95%), and low flow (ΔQ05%) observed under climate change summarized by each USGS gauges location in the ACT River Basin.

| Unique ID | USGSID | Drainage Area  (sq. km) | Change in streamflow (%) | | | | | |
| --- | --- | --- | --- | --- | --- | --- | --- | --- |
|  |  |  | Winter | Spring | Summer | Fall | Q05 | Q95 |
| A0001 | USGS02380500 | 611 | −0.79 | 0.88 | −0.97 | −1.53 | −7.31 | 2.31 |
| A0002 | USGS02381600 | 26 | −1.23 | 1.03 | −0.62 | −1.34 | −6.94 | 1.77 |
| A0007 | USGS02383500 | 2,152 | −0.71 | 1.36 | −0.07 | −1.23 | −5.20 | 2.60 |
| A0008 | USGS02384500 | 653 | −0.06 | 0.97 | 3.91 | 0.47 | −7.33 | 3.02 |
| A0009 | USGS02384540 | 21 | 0.04 | 1.23 | 4.26 | 1.22 | −19.68 | 2.95 |
| A0010 | USGS02385170 | 456 | −1.06 | 1.75 | 8.76 | 0.25 | −3.39 | 2.36 |
| A0011 | USGS02385500 | 104 | −0.86 | 2.16 | 7.46 | 1.07 | −2.42 | 2.50 |
| A0012 | USGS02385800 | 166 | −0.42 | 1.20 | 2.13 | 0.43 | −10.30 | 2.51 |
| A0013 | USGS02387000 | 1,779 | −0.40 | 1.40 | 5.13 | 0.15 | −4.28 | 3.05 |
| A0014 | USGS02387500 | 4,149 | −0.57 | 1.42 | 1.84 | −0.71 | −4.55 | 2.99 |
| A0016 | USGS02388300 | 38 | 1.20 | 3.39 | 9.38 | 5.23 | 2.37 | 4.10 |
| A0017 | USGS02388320 | 43 | 0.86 | 3.18 | 8.77 | 4.81 | −0.14 | 4.01 |
| A0018 | USGS02388350 | 580 | 0.09 | 2.57 | 8.08 | 2.68 | −2.16 | 3.26 |
| A0019 | USGS02388500 | 5,478 | −0.48 | 1.71 | 2.75 | −0.22 | −3.69 | 3.29 |
| A0023 | USGS02390000 | 231 | −0.68 | 1.49 | −0.23 | −2.15 | −5.68 | 1.94 |
| A0029 | USGS02392000 | 1,588 | −0.51 | 1.58 | −0.48 | −1.18 | −5.96 | 2.33 |
| A0031 | USGS02392780 | 360 | 0.50 | 1.70 | −2.26 | 1.09 | −7.40 | 2.42 |
| A0038 | USGS02395120 | 86 | 1.82 | 3.19 | 0.90 | −1.01 | 1.93 | 2.96 |
| A0042 | USGS02397410 | 169 | 2.78 | 3.69 | 2.86 | 5.69 | −5.22 | 4.58 |
| A0043 | USGS02397500 | 298 | 2.58 | 3.57 | 2.82 | 5.22 | −4.94 | 4.43 |
| A0044 | USGS02398000 | 497 | −0.50 | 2.54 | 8.51 | 3.10 | −0.19 | 2.96 |
| A0046 | USGS02398300 | 948 | 0.02 | 2.98 | 11.34 | 5.28 | 2.16 | 3.83 |
| A0047 | USGS02399200 | 515 | −0.06 | 3.08 | 11.49 | 7.64 | 2.25 | 4.20 |
| A0048 | USGS02400100 | 653 | 2.63 | 3.35 | 4.43 | 7.02 | −2.50 | 4.89 |
| A0049 | USGS02400680 | 143 | −0.60 | 3.10 | 9.46 | 8.07 | −0.21 | 4.03 |
| A0050 | USGS02401000 | 471 | −0.61 | 2.84 | 7.00 | 6.92 | −0.64 | 3.66 |
| A0051 | USGS02401370 | 117 | 0.26 | 4.65 | 5.12 | 10.72 | −4.63 | 4.30 |
| A0052 | USGS02401390 | 365 | 0.54 | 4.46 | 5.77 | 10.45 | −4.45 | 4.19 |
| A0055 | USGS02403310 | 495 | 3.73 | 4.54 | 6.20 | 10.95 | −2.32 | 5.75 |
| A0056 | USGS02404400 | 1,246 | 3.32 | 4.95 | 6.12 | 10.30 | −2.57 | 6.25 |
| A0057 | USGS02405500 | 500 | 1.77 | 5.51 | 6.69 | 12.41 | −3.76 | 5.41 |
| A0058 | USGS02406500 | 388 | 2.93 | 5.46 | 6.93 | 11.22 | −2.43 | 6.41 |
| A0060 | USGS02407514 | 368 | 3.01 | 6.09 | 6.67 | 10.60 | −1.75 | 6.33 |
| A0061 | USGS02408540 | 681 | 3.74 | 5.90 | 6.66 | 11.54 | −1.18 | 7.88 |
| A0062 | USGS02410000 | 13 | 3.90 | 8.92 | 9.18 | 13.55 | 0.77 | 9.68 |
| A0063 | USGS02411000 | 25,900 | 1.24 | 3.77 | 4.50 | 4.82 | −1.91 | 5.58 |

**Table S3.** Continued.

| **Unique ID** | **USGSID** | **Drainage Area**  **(sq. km)** | **Change in streamflow (%)** | | | | | |
| --- | --- | --- | --- | --- | --- | --- | --- | --- |
|  |  |  | **Winter** | **Spring** | **Summer** | **Fall** | **Q05** | **Q95** |
| A0065 | USGS02411930 | 704 | 3.45 | 3.96 | 1.70 | 5.82 | −2.17 | 5.66 |
| A0066 | USGS02412000 | 1,160 | 3.69 | 3.81 | 2.13 | 6.47 | −1.63 | 5.94 |
| A0068 | USGS02413210 | 635 | 3.14 | 3.58 | 2.03 | 6.73 | −2.02 | 5.59 |
| A0069 | USGS02413300 | 1,052 | 3.26 | 3.56 | 2.12 | 7.07 | −2.00 | 5.99 |
| A0070 | USGS02414500 | 4,338 | 3.56 | 4.10 | 2.76 | 7.84 | −2.49 | 6.91 |
| A0072 | USGS02414715 | 5,330 | 3.48 | 4.49 | 3.11 | 8.16 | −2.36 | 6.89 |
| A0073 | USGS02415000 | 492 | 3.76 | 5.66 | 6.83 | 11.13 | −4.01 | 7.08 |
| A0074 | USGS02418230 | 185 | 2.69 | 6.92 | 7.40 | 11.37 | 0.03 | 7.95 |
| A0076 | USGS02418760 | 119 | 3.33 | 6.33 | 6.98 | 12.24 | −0.39 | 7.22 |
| A0077 | USGS02419000 | 862 | 4.57 | 7.82 | 9.14 | 16.07 | 0.82 | 9.13 |
| A0078 | USGS02419890 | 12,033 | 3.87 | 6.42 | 6.31 | 11.46 | −0.76 | 8.91 |
| A0080 | USGS02420000 | 38,850 | 2.04 | 4.66 | 5.30 | 6.91 | −1.68 | 6.76 |
| A0081 | USGS02421000 | 751 | 4.37 | 9.68 | 12.95 | 20.45 | 1.97 | 10.66 |
| A0083 | USGS02422500 | 526 | 5.17 | 8.51 | 11.09 | 17.98 | 1.99 | 11.06 |
| A0084 | USGS02423130 | 51 | 0.28 | 5.36 | 5.39 | 9.33 | −5.48 | 4.45 |
| A0085 | USGS02423380 | 363 | 0.80 | 5.42 | 5.46 | 9.69 | −2.47 | 4.88 |
| A0086 | USGS02423400 | 63 | 1.70 | 5.85 | 5.65 | 7.55 | −2.30 | 4.92 |
| A0087 | USGS02423425 | 521 | 1.04 | 5.53 | 5.40 | 9.26 | −2.76 | 5.14 |
| A0088 | USGS02423555 | 868 | 1.43 | 5.85 | 5.11 | 9.05 | −2.65 | 5.82 |
| A0089 | USGS02424000 | 2,660 | 2.15 | 6.88 | 7.98 | 11.86 | −1.32 | 6.76 |
| A0090 | USGS02424590 | 3,833 | 2.43 | 7.23 | 9.29 | 13.24 | −1.26 | 7.32 |
| A0091 | USGS02424940 | 570 | 3.76 | 8.74 | 12.96 | 19.58 | −0.67 | 8.85 |
| A0092 | USGS02425000 | 4,574 | 2.59 | 7.41 | 9.83 | 14.13 | −1.32 | 7.70 |
| A0094 | USGS02427250 | 676 | 3.39 | 9.00 | 14.10 | 23.29 | 3.93 | 8.01 |
| A0095 | USGS02427700 | 253 | 2.66 | 10.05 | 18.01 | 23.22 | −1.49 | 8.38 |
| A0096 | USGS02428400 | 54,390 | 2.37 | 5.71 | 7.14 | 10.10 | −0.63 | 7.58 |

# References

1 Ashfaq, M. *et al.* High-resolution ensemble projections of near-term regional climate over the continental United States. *Journal of Geophysical Research: Atmospheres* **121**, 9943-9963, doi:10.1002/2016jd025285 (2016).

2 Ashfaq, M., Bowling, L. C., Cherkauer, K., Pal, J. S. & Diffenbaugh, N. S. Influence of climate model biases and daily‐scale temperature and precipitation events on hydrological impacts assessment: A case study of the United States. *Journal of Geophysical Research: Atmospheres (1984–2012)* **115** (2010).

3 Ashfaq, M. *et al.* Near‐term acceleration of hydroclimatic change in the western US. *Journal of Geophysical Research: Atmospheres* **118** (2013).

4 Leavesley, G., Lichty, R., Troutman, B. & Saindon, L. Precipitation-runoff modeling system: User’s manual. *Water-resources investigations report* **83**, 4238 (1983).

5 Markstrom, S. L. *et al.* PRMS-IV, the precipitation-runoff modeling system, version 4. *US Geological Survey Techniques and Methods* (2015).

6 Hay, L. E., Markstrom, S. L. & Ward-Garrison, C. Watershed-Scale Response to Climate Change through the Twenty-First Century for Selected Basins across the United States. *Earth Interactions* **15**, 1-37, doi:10.1175/2010ei370.1 (2011).

7 Najafi, M. R., Moradkhani, H. & Jung, I. W. Assessing the uncertainties of hydrologic model selection in climate change impact studies. *Hydrological Processes* **25**, 2814-2826, doi:10.1002/hyp.8043 (2011).

8 Regan, R. S. *et al.* The US Geological Survey National Hydrologic Model infrastructure: Rationale, description, and application of a watershed-scale model for the conterminous United States. *Environmental modelling & software* (2018).

9 Viger, R. & Bock, A. GIS features of the geospatial fabric for national hydrologic modeling. *US Geological Survey,* [*https://doi*](https://doi)*. org/10.5066/F7542KMD* (2014).

10 Driscoll, J., Markstrom, S., Regan, R., Hay, L. & Viger, R. National Hydrologic Model Parameter Database: 2017‐05‐08 download. *US Geological Survey* (2017).

11 Markstrom, S. L., Hay, L. E. & Clark, M. P. Towards simplification of hydrologic modeling: identification of dominant processes. *Hydrology and Earth System Sciences* **20**, 4655-4671, doi:10.5194/hess-20-4655-2016 (2016).

12 Liang, X., Lettenmaier, D. P., Wood, E. F. & Burges, S. J. A Simple Hydrologically Based Model of Land-Surface Water and Energy Fluxes for General-Circulation Models. *Journal of Geophysical Research-Atmospheres* **99**, 14415-14428, doi:Doi 10.1029/94jd00483 (1994).

13 Lohmann, D., Raschke, E., Nijssen, B. & Lettenmaier, D. Regional scale hydrology: I. Formulation of the VIC-2L model coupled to a routing model. *Hydrological Sciences Journal* **43**, 131-141 (1998).

14 Lohmann, D., NOLTE‐HOLUBE, R. & Raschke, E. A large‐scale horizontal routing model to be coupled to land surface parametrization schemes. *Tellus A* **48**, 708-721 (1996).

15 Brakebill, J. W., Wolock, D. M. & Terziotti, S. Digital hydrologic networks supporting applications related to spatially referenced regression modeling. *JAWRA Journal of the American Water Resources Association* **47**, 916-932 (2011).

16 Naz, B. S. *et al.* Regional hydrologic response to climate change in the conterminous United States using high-resolution hydroclimate simulations. *Global and Planetary Change* **143**, 100-117, doi:10.1016/j.gloplacha.2016.06.003 (2016).

17 Oubeidillah, A. A., Kao, S. C., Ashfaq, M., Naz, B. S. & Tootle, G. A large-scale, high-resolution hydrological model parameter data set for climate change impact assessment for the conterminous US. *Hydrology and Earth System Sciences* **18**, 67-84, doi:10.5194/hess-18-67-2014 (2014).

18 VanShaar, J. R., Haddeland, I. & Lettenmaier, D. P. Effects of land-cover changes on the hydrological response of interior Columbia River basin forested catchments. *Hydrological Processes* **16**, 2499-2520, doi:10.1002/hyp.1017 (2002).

19 Cuo, L., Lettenmaier, D. P., Alberti, M. & Richey, J. E. Effects of a century of land cover and climate change on the hydrology of the Puget Sound basin. *Hydrological Processes* **23**, 907-933, doi:10.1002/hyp.7228 (2009).

20 Storck, P., Bowling, L., Wetherbee, P. & Lettenmaier, D. Application of a GIS‐based distributed hydrology model for prediction of forest harvest effects on peak stream flow in the Pacific Northwest. *Hydrological Processes* **12**, 889-904 (1998).

21 Wigmosta, M. S., Nijssen, B., Storck, P. & Lettenmaier, D. The distributed hydrology soil vegetation model. *Mathematical models of small watershed hydrology and applications*, 7-42 (2002).

22 Wigmosta, M. S., Vail, L. W. & Lettenmaier, D. P. A distributed hydrology‐vegetation model for complex terrain. *Water resources research* **30**, 1665-1679 (1994).

23 Gangrade, S. *et al.* Sensitivity of Probable Maximum Flood in a Changing Environment. *Water Resources Research* **54**, 3913-3936, doi:10.1029/2017wr021987 (2018).
